# Genomic architecture of phenotypic extremes in a wild cervid

**DOI:** 10.1186/s12864-022-08333-x

**Published:** 2022-02-12

**Authors:** S. J. Anderson, S. D. Côté, J. H. Richard, A. B. A. Shafer

**Affiliations:** 1 Environmental and Life Sciences Graduate Program, Trent University, Peterborough, K9J 7B8 Canada; 2 Département de biologie, Centre d’études nordiques & Chaire de recherche industrielle CRSNG en aménagement intégré des ressources de l’île d’Anticosti, Université Laval, Québec, QC G1V 0A6 Canada; 3 Forensics Department, Trent University, Peterborough, K9J 7B8 Canada

**Keywords:** Quantitative traits, Population genetics, Gene enrichment, Pool-seq, Extreme phenotypes

## Abstract

Identifying the genes underlying fitness-related traits such as body size and male ornamentation can provide tools for conservation and management and are often subject to various selective pressures. Here we performed high-depth whole genome re-sequencing of pools of individuals representing the phenotypic extremes for antler and body size in white-tailed deer (*Odocoileus virginianus*). Samples were selected from a tissue repository containing phenotypic data for 4,466 male white-tailed deer from Anticosti Island, Quebec, with four pools representing the extreme phenotypes for antler and body size after controlling for age. Our results revealed a largely homogenous population but detected highly divergent windows between pools for both traits, with the mean allele frequency difference of 14% for and 13% for antler and body SNPs in outlier windows, respectively. Genes in outlier antler windows were enriched for pathways associated with cell death and protein metabolism and some of the most differentiated windows included genes associated with oncogenic pathways and reproduction, processes consistent with antler evolution and growth. Genes associated with body size were more nuanced, suggestive of a highly complex trait. Overall, this study revealed the complex genomic make-up of both antler morphology and body size in free-ranging white-tailed deer and identified target loci for additional analyses.

**Supplementary Information:**

The online version contains supplementary material available at 10.1186/s12864-022-08333-x.

## Background

Characterizing the genomic architecture underlying phenotypes in natural populations provides insights into the evolution of quantitative traits [1]. Some quantitative traits are correlated with metrics of fitness and are particularly important because they might directly influence population viability [2]. This relationship between genomic architecture and quantitative traits, however, is not easy to empirically identify [3], and often has unclear and unpredictable responses to selection [4]. Sample size in particular, of both the number of sequenced individuals and assayed SNPs, greatly limits the power of genome scans [5]. As such, alternative sequencing and sampling strategies have emerged to identify the genetic basis of traits in natural populations.

One approach that has gained traction seeks to sample individuals representing the extreme ends of the spectrum for any observable phenotype, instead of randomly sampling individuals from the entire distribution (e.g. [5–10]). This sampling methodology of so-called extreme phenotypes aims to maximize the additive genetic variance for the sampled trait, increasing the power to detect quantitative trait loci or QTL [5]. Simulations have showed that sampling the whole genome increases power [11], but this varies by the effect size of each SNP [5]. Population history (i.e. degree of linkage disequilibrium) also has a profound effect on identifying QTL, essentially making the ability to identify linked or causative loci easier in small (isolated) populations, and challenging in large populations with high effective population size (*N*_e_), and by proxy high recombination rates (see also [12]).

In contrast to individual genome re-sequencing, pooled sequencing (pool-seq) uses DNA from many individuals within a population and is a cost-effective alternative to the whole genome sequencing of each individual separately [13]. Individual identity is lost through the pooling of DNA, but the resulting allelic frequencies are representative of the population and can be used to conduct standard population genomic analyses, including QTL mapping and association studies [14]. Pool-seq methods have successfully identified genes underlying colour morphs in butterflies and birds, abdominal pigmentation in *Drosophila*, horn size of wild Rocky Mountain bighorn sheep, and growth and reproduction in salmon [15–18]. Depending on the quality of annotation, similar pooled approaches can characterize differences in transposable element insertions [19]. The latter aspect, broadly speaking, is particularly relevant for genotype-phenotype correlations as most causative SNPs occur outside coding regions [3, 20], and there is evidence of TE insertions causing phenotypic differences [21] that are adaptive [22].

Combining pool-seq and sampling of phenotypic extremes seems promising, if not only for financial considerations [23], in studies interested in the genetic basis of traits in wild populations. Coalescent simulations by Hivert et al. [24] showed high accuracy of *F*_ST_ estimates from pool-seq, at times more precise than individual genomes. Similarly, Inbar et al. [25] showed that pooled approaches produced near identical allele frequency distributions to that of whole and reduced genome sequencing, with simulations reliably detecting moderate to large effect QTL. While Bastide et al. [26] showed that low frequency alleles and small effect sizes, particularly for complex traits, were difficult to detect, this is also the case for non-pooled studies (e.g. Caballero et al. [11]). Recent pool-seq work by Mohamed et al. [23] and Michelletti and Narum [17] identified the same associated regions for traits previously identified with extensive SNP arrays [27] and pedigree-based QTL mapping [28], respectively. Even when effect sizes are small, gene set and pathway analyses can be used to detect the collective effect of SNPs underling the traits [29, 30], and provide complementary tools to detect genetic associations to a given phenotype [31].

Here, we explored the genomic basis for phenotypic variation by sampling the extreme distribution ends of a phenotype in a non-model big-game species, the white-tailed deer (*Odocoileus virginianus*, WTD). Two traits are of particular interest in WTD; body size and antler size, which have a degree of observable variation [32] and are connected to individual reproductive success [33–35]. Heritability for antler and body measurements are moderate to high for antler features and body size [36–38]. Large antlers and body size are also sought after by hunters, both as trophies and food throughout North America. Our objective was to identify genomic windows and TE insertions associated with variation in antler and body size phenotypes in WTD; we assume a polygenic trait (e.g. [39]), but due to extreme phenotypic sampling there should be detectable outlier windows and gene pathways related to these phenotypes. This to our knowledge the first application of pooled-GWAS study of extreme phenotypes in a free-ranging population.

## Methods

### Study area

Anticosti Island (49°N, 62°W; 7,943 km^2^) is located in the Gulf of St. Lawrence, Québec (Canada) at the northeastern limit of the white-tailed deer range (Fig. S1). The island is within the balsam fir-white birch bioclimatic region with a maritime sub-boreal climate characterized by cool and rainy summers (630mm/year), and long and snowy winters (406 cm/year; Environment [40, 41]). Approximately 220 white-tailed deer were introduced between 1896 and 1897 and the population has an estimated contemporary *N*_e_ of ~1,500 [42]: the island’s demographic history might have resulted in increased additive variance [43]. There is no population subdivision [42] and the mean relatedness of our database is zero [36]. Any related individuals in the data set should manifest in genome-wide patterns as opposed divergent windows between pools. Quantitative genetic analyses showed no effect of sampling year on trait heritability, which was interpreted as a uniform environmental effect across the island [36].

### Sample Collection and Phenotypic Distribution

No live animals were directly involved in this study, rather we collected tissue samples stored in 95% ethanol and phenotypic data on 4,466 male deer harvested by hunters from September to early December, 2002–2014 on Anticosti Island. The sample and measurements were completed as part of a large effort led by the NSERC Industrial Research Chair in integrated resource management of Anticosti Island focussed on collecting data on the body condition of deer on the island. We used cementum layers in incisor teeth to age individuals [44]. Two metrics of antler size were selected: the number of antler tines or points (>2.5 cm) and beam diameter (measured at the base; ±0.02 cm) [45]. We used one metric for body size: body length which is correlated to other metrics such as body weight and hind foot length [46]. Because antler and body size of male cervids are correlated to age [47, 48], we used linear models to first assess the relationship between age and each metric separately. We then computed an antler and body size index based on the average rank of each individual’s residual variation for the phenotypic metrics. The top and bottom ranked individuals from each group were selected from the available database for DNA extraction, with only non-overlapping samples for large and small phenotypes from the same year, were included in the final pool to limit temporal variation. This created four groups for pooled-sequencing: large antler (LA), small antler (SA), large body size (LB), and small body size (SB; Table S1).

### DNA Extraction and Genome Sequencing

We isolated DNA from tissue using the Qiagen DNeasy Blood & Tissue Kit. The concentration of each DNA extract was determined using a Qubit dsDNA HS Assay Kit (Life Technologies, Carlsbad, CA, USA). We aimed to sequence each pool to 50X as per recommendations of Schlötterer et al. [14]. Equal quantities of DNA (100 ng/sample) were combined into representative pools for LA (*n* = 48), and SA (*n* = 48), LB (*n* = 54), and SB (*n* = 61) for a desired final concentration of 20 ng/ul combined DNA for each pool. Sequencing was conducted at The Centre for Applied Genomics (Toronto, ON, Canada) on an Illumina HiSeqX with 150 bp pair-end reads (Table S2).

### Genome Annotation

The draft WTD genome constructed from long (PacBio) and short-read (Illumina) data was used as a reference (NCBI PRJNA420098; Accession No. JAAVWD000000000). We performed a full genome annotation by masking repetitive elements throughout the genome using a custom WTD database developed through repeat modeler v1.0.11 [49] in conjunction with repeatmasker v4.0.7 [50] using the non-masked NCBI repeat database for artiodactyla, without masking low complexity regions. The masked genome was annotated using the MAKER2 v2.31.9 pipeline [51]. We used a three-stage process [52] for the generation of an initial training data set; 1) using publicly available white-tailed deer EST and Protein sequences available through NCBI an initial GFF file was generated with SNAP v2013-11-29 [53]; 2) an hmm file was generated from the initial SNAP training GFF output and was used as evidence for the MAKER2 prediction software, again using SNAP; and lastly 3) evidence from the prior SNAP trials in GFF format were used as for the generation of a training data set for AUGUSTUS v3.3.2 gene prediction software. Gene IDs were generated using blastp v2.9.0 [54] on the WTD annotation protein transcripts, restricting the blast search to human protein annotations in the uniport database (parameters -max_hsps 1 -max_target_seqs 1 -outfmt 6 -taxids 9606).

### Mapping and SNP calling

We trimmed reads for quality and adaptors using the default Trimmomatic v.0.36 settings [55]. Reads were then aligned to the unmasked WTD reference genome with BWA-mem v0.7.17 [56]. We used samtools v1.10 [57, 58] to merge and sort all aligned reads into four files for each representative pool. Prior to calling SNPs we filtered for duplicates using Picard v2.20.6 [59], kept uniquely mapped reads with samtools, and conducted local realignment using GATKv3.8 [60]. We called SNPs with samtools mpileup (minimum mapping quality (-q) = 20). INDELs were identified using a perl script in the Popoolation2 software suite [61, 62], and all INDELs as well as SNPs within 5 kb up/downstream of these regions were removed. We filtered out all masked regions and all scaffolds <= 50 kb.

Genome wide differences were calculated between large and small phenotypes for antler and body size. We used a sliding window approach with window sizes of 1000 bp with a step size of 500 bp and minimum covered fraction of 0.8 to test for allele frequency differences using Fisher’s exact test (FET) and estimate the fixation index (*F*_ST_) from the Popoolation2 software suite [61, 62]. A minimum and maximum coverage were determined based on the mode depth +/- half the mode as per Kurland et al. [63] and a minimum overall count of the minor allele of 3 for each pool was specified (Fig. S2). We calculated the mean allele frequency differences of each window in R v3.6.1. We first corrected for multiple testing by using an FDR correction (see [64]) but also set a conservative α (1.0e-7) as Popoolation multiples all *p*-values within each window by default. We ran all subsequent analyses on the FET windows, noting here a correlation to *F*_ST_ (Fig. S3). Manhattan plots were generated using a custom R script to plot the distribution of outliers by position and scaffold throughout the entire WTD genome. Bedtools v2.27.1 was used to characterize whether a window was within 25 kb up/downstream of a gene (i.e. regulatory) which were used for gene set analysis (below). To identify the most differentiated SNPs within each outlier region we used the modified chi-square test from Spitzer et al. [65] that accounts for overdispersion. This multi-pronged approach to identify outlier windows and SNPs was used to limit the potential for false positives.

Lastly, we generated two null populations to quantify the rate of false positives. Here, we combined all the reads from SA, SB, LA, and LB into one large pool using samtools -merge; we then generated two artificial pools by randomly sub setting reads using the samtools view -s flag that matched the coverage of our original pools. The same window-based analyses were conducted assuming any outlier would represent background noise. Note that pool-seq uses read counts to estimate allele frequencies, hence the need for high coverage; sampling variance is expected to generate some differences in minimally diverged populations as for example 50X coverage with a minor allele count of 3 and 4 results in a 2% allelic frequency difference.

### Transposable Elements

We used consensus transposable element (TE) sequences from the repbase database for cow to repeat mask the WTD reference genome, and subsequently merged this masked genome with the repbase TE reference sequences [66]. We used the repbase database consensus sequences to re-mask the genome due to more robust information for TE identities, family and order required in this analysis. Trimmed reads for both antler and body size phenotypes were aligned to the TE-WTD merged reference genome using bwa-mem as stated in previous methods for SNP analysis. We used the recommended workflow for the software suite popoolationTE2 v1.10.04 [19] to create a list of predicted TE insertions. From this we calculated TE frequency differences between large and small phenotypes as well as proximity to genic regions. Here, we only examined TEs that were within 25 kb up or downstream from a gene and within the 95^th^ percentile for absolute frequency differences to allow for more features to be assessed subsequently. The same analyses were performed on our null pools to generate the null TE frequency distribution.

### GO Pathways

To evaluate the enrichment of gene pathways in our outlier windows the program Gowinda v1.12 [67] was used to test for GO term enrichment, while also accounting for biases in gene length but requires input of individual SNPs. We also used TE start position as a proxy for SNP location for program compatibility. A gtf version of our annotation was created by removing duplicate genes and retaining only the longest transcript – resulting in 15,395 unique genes. All SNPs in outlier windows that were within 25 kb of a gene were included in this analysis and compared to all SNPs in every outlier window for antler and body size phenotypes independently. Similarly, all TEs that were within 25 kb up or downstream from a gene and within the 95th percentile for absolute frequency differences were included in a separate analysis for antler and body phenotypes independently. We used the program REVIGO [68] to remove redundant GO terms and to visualize semantic similarity-based scatterplots: the latter turns GO biological descriptions into numerical values that allow for aggregating similar terms. Results from Gowinda with *p*-values < 0.01 were used with REVIGO to generate plots of significant GO terms for biological processes of antler and body size phenotypes. Only GO terms with a dispensability score < 0.20 were included in figures, representing the least redundant terms in the analysis.

We also created outlier gene ID lists (i.e. genes within 25 kb of an outlier TE and window) for antler and body size that were provided independently to DAVID v6.8 [69], while removing all duplicate gene IDs. DAVID uses a modified FET to determine the significance of enrichment for any given GO pathway based on the size of the gene list provided, the number of genes used as a background for a species, and level of enrichment for each term from the list. Results from DAVID with a *p*-value < 0.01 were used with REVIGO to generate plots of significant GO terms for biological processes, molecular function, and cellular components of antler and body size phenotypes.

### Validation of outliers

Pool-seq SNP callers can vary in their allele frequency estimates [70]; we therefore selected three candidate SNPs for qPCR validation of allele frequencies. The SNPs were selected based off significant FET and chi-square values, were near or inside a gene, and passed quality control with respect to primer design (Fig. S4 & S5). Custom genotyping assays using rhAMP chemistry (Integrated DNA Technologies) were designed and genotyped on the QuantStudio 3 (Thermo Fisher Scientific). Oligo sequences and reaction parameters are provided in Table S3. Using the phenotypic category as the binary response variable, we ran a logistic regression treating the genotypic data as additive (e.g. 0-2 LA/LB alleles per locus, per individual) to calculate the effect size.

## Results

### Phenotypes

**Fig. 1:**
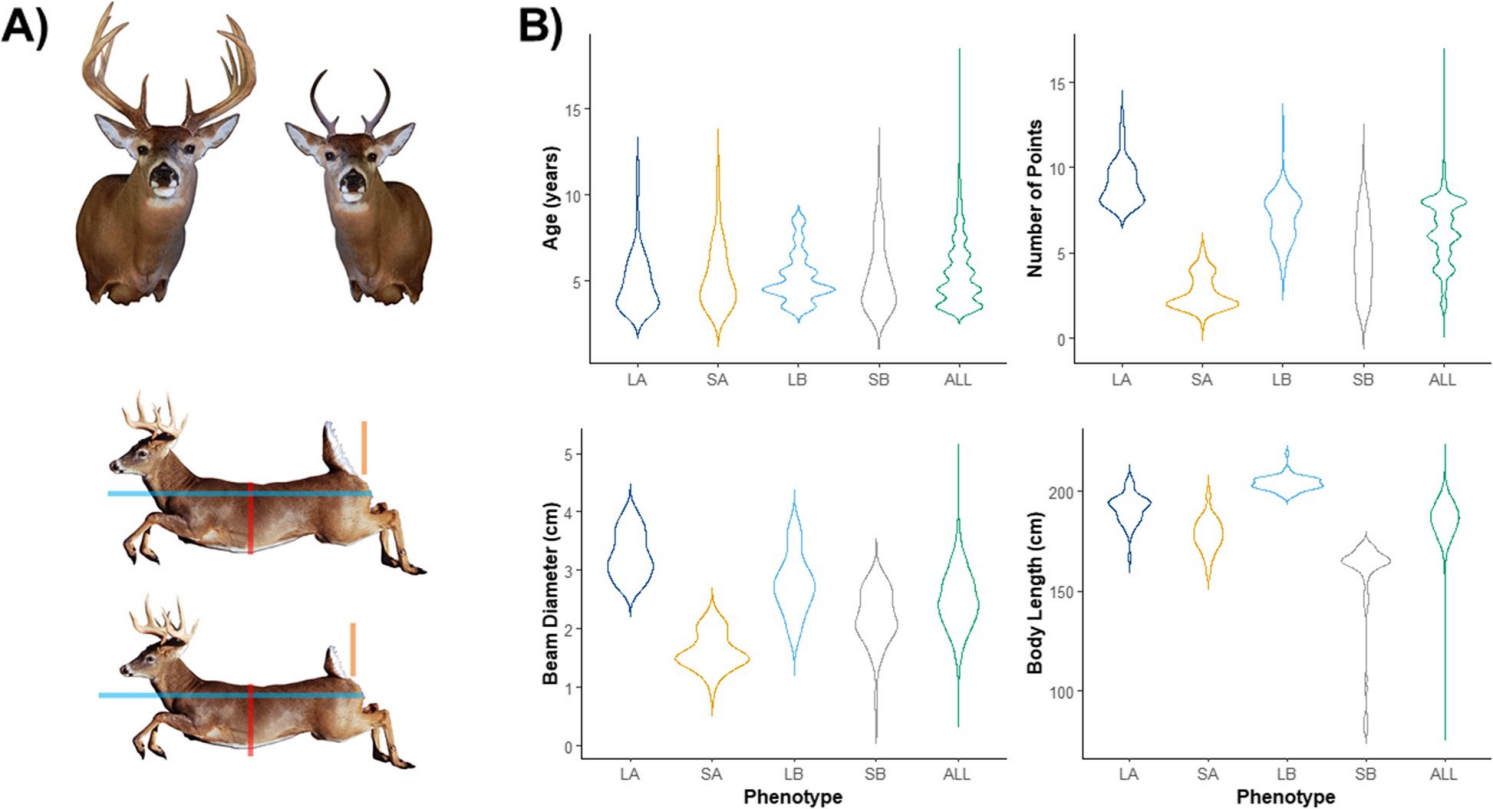
The phenotypic extremes used in our sampling methodology. **a** Artist renderings of the average phenotypic measures for all individuals included in each pool. Measurements for antler pools (top) included mainbeam diameter and number of points, while body size (bottom) included body length. Scale bars are present for body size to better show differences in average body length, and chest circumference between large and small phenotypes. **b** Violin plots displaying the original measurements of each individual, grouped by large antler (LA), small antler (SA), large body size (LB), and small body size (SB) pools. The phenotypes measured include age (years), number of points, beam diameter (cm), and body length (cm)

We selected the top individuals at the tail ends of the distribution for measurements used in our antler and body size rankings which are representative of the extreme phenotypes. Artist renderings and the distribution of measurements for the number of antler points, beam diameter, and body length between the groups of individuals representing each extreme phenotype pool (LA, SA, LB, SB) are shown in Fig. 1a. There were no significant differences in mean age between the pools (Fig. 1b) and individual rankings between antler and body size were weakly correlated (Pearson *r* = 0.33, *p* <0.01).

### Detecting genetic variants and their genomic regions

**Fig. 2:**
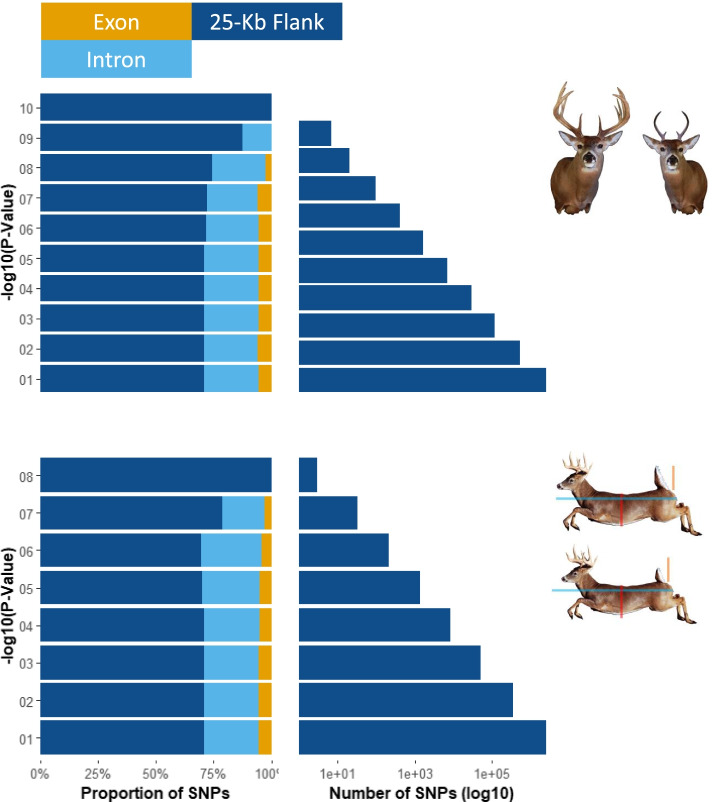
Stacked barplots representing the proportion of SNPs from all windows in different genomic regions (exon, intron, or 25 kb flank). Each bin is grouped by -log10(*p*-values) calculated by Fisher’s Exact Test. and corresponds with the y-axis values from the Manhattan plots. Coordinating barplots represent the number of SNPs in each bin is presented on a log scale

**Fig. 3:**
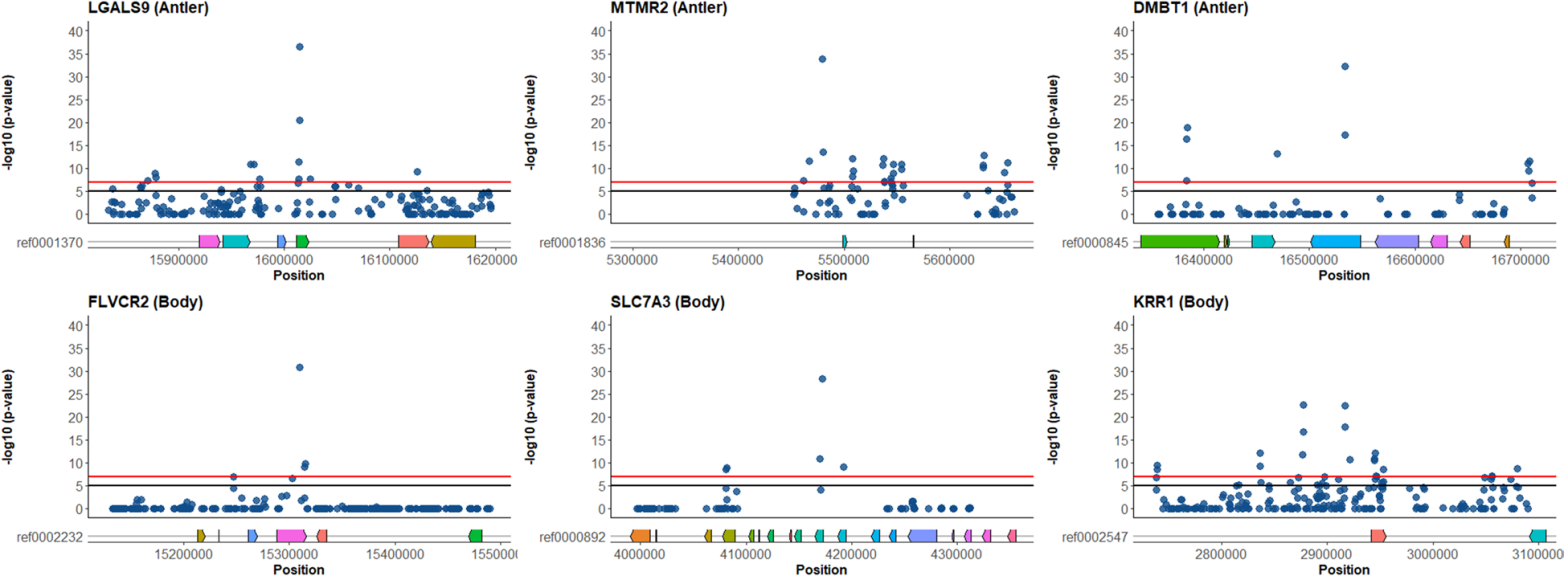
Manhattan plots representing pairwise genetic differentiation (Fisher’s Exact Test) for scaffolds of interest in 1000 bp sliding windows, with a step size of 500. Plots represent 200 kbp (+/- ) surrounding the most highly differentiated win dows and their associated gene regions; the top panel representing *LGALS9* (ref0001370), *MTMR2* (ref0001836), and *DMBT1* (ref0000845) from the comparison of antler phenotype pools and the bottom panel representing *FLVCR2* (ref0002232), *SLC7A3* (ref0000892), and *KRR1* (ref0002547) from the comparison of body size phenotype pools. The horizontal black like represents the false detection rate (*p *= 10^-5^), while the red line represents a conservative significance threshold (*p *= 10^-7^)

The total reads generated from resequencing are observed in Table S 2 (BioProject ID PRJNA576136). The WTD genome is 2.52 GB and has a N50 of 17 MB, 727 scaffolds >50 kb (98.85% of genome), and BUSCO completeness of 91% across 303 orthologs. The genome annotation resulted in 20,750 genes and we identified 29,484,233 and 29,703,634 SNPs from the antler and body analyses respectively across the genome, with 25,116,953 shared SNPs (Fig. 2). The mean allele frequency difference across the antler outlier windows were calculated to be 14% with 5038 windows meeting the FDR and α threshold (*p* <= 1x10^-7^); many of the top windows were adjacent to genes with known functions (Fig. 3; Tables 1 & 2; full gene list in Tables S4 & S5). For the comparison of body size, the mean allele frequency difference was 13% in 1301 windows meeting the FDR threshold. 247 windows overlapped between phenotypes. For all individual SNPs within outlier windows the modified chi-square test with FDR correction identified 5,184 and 1,166 divergent SNPs (*p* < 0.01) from the antler and body analysis, respectively. Overall genome-wide allele frequency differences in both phenotype pools were 8%; in contrast, the null pool comparisons had 3% difference on average and only 5 windows met the FDR cut-off with a mean allele frequency difference of 5%.

**Table 1:** The top 10 outlier window positions from the antler trait analysis based on corrected *p*-values that were within 25 kb of genes or regulatory regions up/downstream. The closest gene is listed for each window. Functions of each gene are abbreviated from RefSeq/Uniprot

Scaffold	Window Position	***p***	Gene	Function
ref0001370	16015000	2.66E-37	LGALS9	The galectins are a family of beta-galactoside-binding proteins implicated in modulating cell-cell and cell-matrix interactions.
ref0001836	5479500	1.24E-34	MTMR2	This gene is a member of the myotubularin family of phosphoinositide lipid phosphatases.
ref0000845	16534000	5.97E-33	DMBT1	Considered as a tumor suppressor gene. May play a roll in epithial differentiation.
ref0002391	7908000	1.75E-29	ITLN2	ITLN2 (Intelectin 2) is likely involved include carbohydrate binding.
ref0001924	2123500	1.52E-28	TRIM64	TRIM proteins are involved in pathogen-recognition and by regulation of transcriptional pathways in host defence (Ozato et.al., 2008).
ref0000845	16751500	1.14E-27	CD163	Involved in the clearence and endocytocis of hemoglobin/heptaglobin complexes by macrophages. May protext tissues from free hemoglobin-mediated oxidative damage.
ref0002391	7907500	1.79E-27	ITLN2	See above.
ref0000505	9079500	1.37E-26	ATP5F1A	This gene encodes a subunit of mitochondrial ATP synthase.
ref0000881	8059500	1.91E-26	MYH9	This gene encodes a conventional non-muscle myosin.
ref0002644	7312000	2.23E-26	RIMS1	The protein encoded by this gene is a RAS gene superfamily member that regulates synaptic vesicle exocytosis.

**Table 2:** The top 10 outlier window positions from the body trait analysis based on corrected *p*-values that were within 25 kb of genes or regulatory regions up/downstream. The closest gene is listed for each window. Functions of each gene are abbreviated from RefSeq/Uniprot

Scaffold	Window Position	***p***	Gene	Function
ref0002232	15310000	1.71E-31	FLVCR2	This gene encodes a transmembrane protein that is a calcium transporter.
ref0000892	4172500	4.82E-29	SLC7A3	SLC7A3 appears to be involved in amino acid transmembrane transporter activity and basic amino acid transmembrane transporter.
ref0002547	2917000	2.98E-23	KRR1	KRR1 (KRR1 Small Subunit Processome Component Homolog) appears to be involved win cytosol and Gene Expression.
ref0000845	16369000	1.26E-20	CD163	Involved in the clearence and endocytocis of hemoglobin/heptaglobin complexes by macrophages. May protext tissues from free hemoglobin-mediated oxidative damage.
ref0000438	13320500	1.64E-20	KIF3C	Microtubule-based anterograde translocator for membranous organelles.
ref0002813	16028000	8.08E-20	DOCK1	This gene encodes a member of the dedicator of cytokinesis protein family.
ref0002788	19947500	2.45E-19	SYK	This gene encodes a member of the family of non-receptor type Tyr protein kinase that is involved in coupling activated immunoreceptors to downstream signaling events that mediate diverse cellular responses, including proliferation, differentiation, and phagocytosis.
ref0000529	431000	4.31E-19	TRDV1	T cell receptors recognize foreign antigens which have been processed as small peptides and bound to major histocompatibility complex (MHC) molecules at the surface of antigen presenting cells (APC).
ref0002645	24851500	8.41E-19	RNASEH1	Endonuclease that specifically degrades the RNA of RNA-DNA hybrids
ref0002547	2916500	1.27E-18	KRR1	See above.

### TE Insertions

We identified 19,160 TE insertions through the joint analysis of antler phenotype sequences (both large and small). Of these TEs, we identified 950 insertions that fell in the 95th percentile for the absolute difference between large and small phenotypes for further analysis (>|0.20|%; Fig. S6). Of the 95th percentile TE insertions in the antler analysis, 92 were found to overlap with genes, with 275 within a 25 kb window up or downstream of genic regions. For body size, 6,610 TE insertions were identified with 320 insertions in the 95th percentile (>0.21%). Of these, 28 overlapped with genes, and 104 within 25 kb of a genic region. The null pool TE comparisons had considerably shifted frequency distribution, with the 95% percentile being TEs that differed by >0.11%.

### Gene Ontology Annotations

**Fig. 4:**
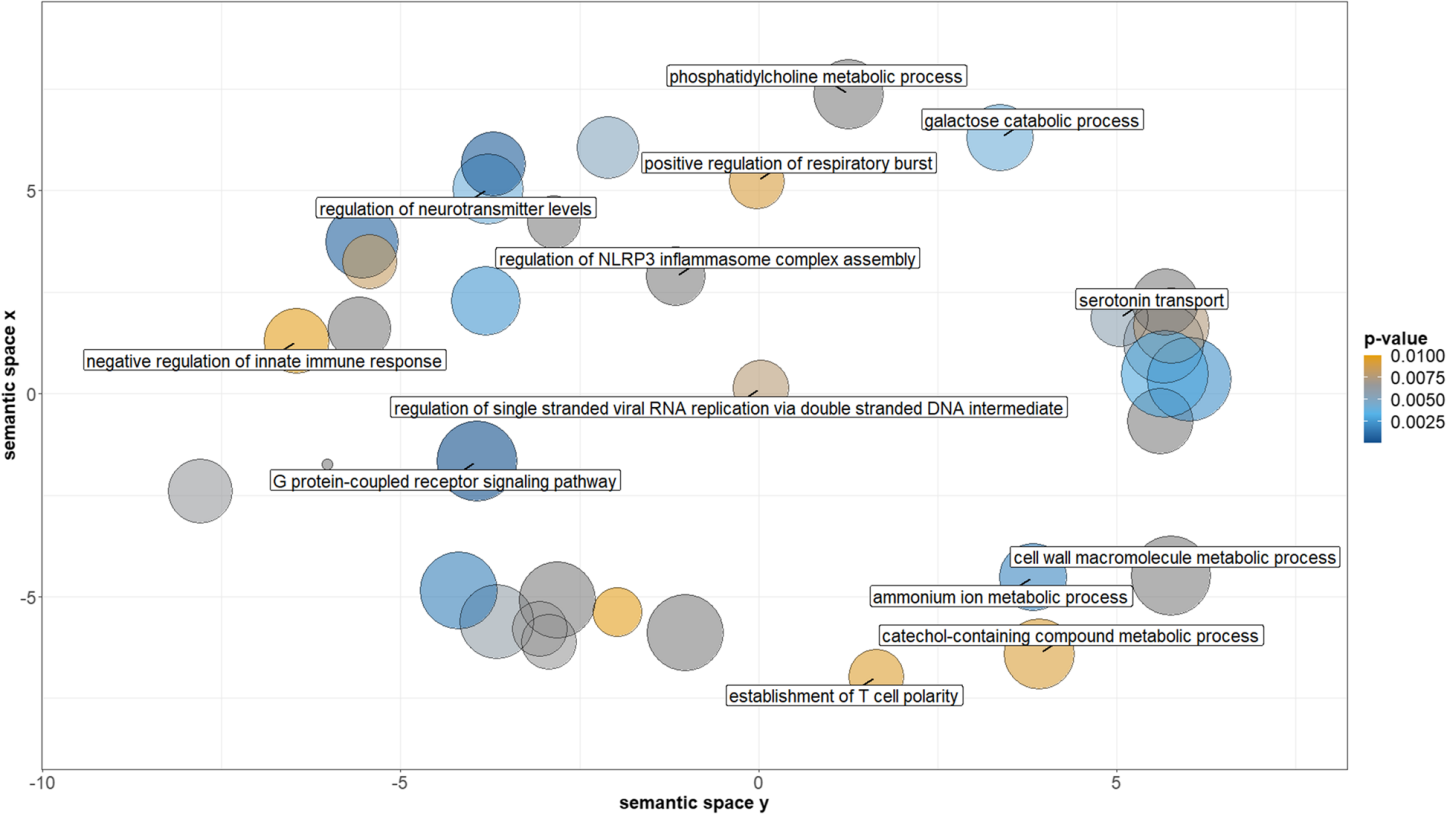
Antler FET analysis of GO terms grouped by semantic similarity. Points are coloured based on significance, with all terms with *p*-values < 0.01 from the output of the Gowinda analysis for gene enrichment being included in the analysis. The size of each point represents the specificity of each term; GO terms for smaller points being more specific, and larger points more general. Only points with a dispensability score < 0.20 are labeled

The results from Gowinda comparing all SNPs within outlier windows to background SNPs from all windows showed the top 10 enriched GO terms and gene counts (Fig. S7). Six statistically enriched GO terms (FDR < 0.01) from the antler analysis were: GO:0004888 (transmembrane signaling receptor activity), GO:0004872 (receptor activity), GO:0038023 (signaling receptor activity), GO:0004930 (G-protein coupled receptor activity), GO:0022835 (transmitter-gated channel activity), GO:0022824 (transmitter-gated ion channel activity). There were no significant GO terms identified through the body analysis following FDR corrections. We show term reductions for significantly enriched GO terms (*p*-value < 0.01) through the program REVIGO, with clustered items relating to the semantic similarity in the antler pool (Fig. 4 and Fig. S7) and body pools (Fig. S8). There were no significant GO terms identified through the TE analysis using Gowinda and REVIGO following FDR corrections (FDR < 0.01) for both the antler and body analysis.

DAVID pathway analysis identified 6 statistically significant GO terms (FDR < 0.05); GO:0006614 (SRP-dependent cotranslational protein targeting to membrane), GO:0000184 (nuclear-transcribed mRNA catabolic process, nonsense-mediated decay), GO:0019083 (viral transcription), GO:0006413 (translational initiation), GO:0055085 (transmembrane transport), and GO:0007586 (digestion). No significant terms were identified through the body analysis. There were insufficient outliers (*n* = 5) with adjacent genes (*n* = 2) to conduct these analyses on the null pools.

### Validation of Outliers

We genotyped putative antler SNPs (*RIMS1*, and *SRP54*) and one body locus (*LIRF1*) split between pools which resulted in 80 and 70 individuals having complete genotype and phenotypic data, respectively, that were a subset of the pooled samples. The allele frequency differences between poolseq and qPCR were as follows: *RIMS1* (15% vs 6%), SRP54 (16% vs 10%), and LIRF1 (12% vs 16%). There was an effect of the number of antler alleles at the *SRP54* locus on antler category (ß = -0.99, *p* = 0.02) and the *LIRF1* alleles had an effect on body size. (ß = -0.94, *p* = 0.03; The RIMS1 locus frequency differences were not shown to have an effect in the model (*p* > 0.05).

## Discussion

The extensive database with phenotypic measurements for Anticosti Island white-tailed deer allowed us to select individuals from the full range of the distribution resulting in a sampling representative of extreme phenotypes (Fig. 1). The deer on the island form a panmictic population and have a smaller *N*_e_ [42] relative to contiguous mainland populations [71]. Moreover, quantitative genetic assessment revealed a consistent environmental effect across traits [36]. Collectively, these factors suggest a pooled-sequencing approach of phenotypic groups could identify key genes and pathways underlying these important traits.

We identified outlier regions for both traits that are atypically differentiated for what would be expected in this population and our null distribution model. The most divergent windows and TEs for antler and body traits are widely dispersed throughout the WTD genome. While effect sizes cannot be estimated from pooled samples, the sampling suggests we have maximized the additive variance of this population and these outlier windows are likely associated with the traits. The GO analysis, and more broadly a collection of small putative islands of divergence (Fig. 3), is consistent with a polygenic trait controlled by many genes: this interpretation is also supported by the literature on body size and ornaments in mammals [72–74], including red deer [39].

### Genomic architecture of antlers and body size

Phenotypic association of SNPs has traditionally focussed on non-synonymous variants, but it is evident that non-coding regions impact phenotypic variation [20], often with clear relationships to promoters and enhancer regions [75–77]. While some outliers are surely false positives [78, 79], cattle GWAS for similar traits typically identify 100s to 1000s of QTL [80, 81]. Our data analysis – including the GO pathway analyses that incorporated TE insertions – suggest that it is the cumulative effects from these variants on biological pathways underlying antler and body trait variation in white-tailed deer.

Antlers are the only completely regenerable organ found in mammals [82], a unique process that involves simultaneous exploitation of oncogenic pathways and tumor suppressor genes, and the rapid recruitment of synaptic and blood vesicles [74]. Antlers might also be an honest signal of male quality [83]. A study of differential RNA expression in sika deer antlers identified numerous gene clusters, and we detected an overlap in gene pathways associated with cell death, cell wall formation, and protein metabolism (Fig. 4 [84];). One notable outlier in our study also detected by Ba et al. [84] is *IGF1R* that is responsible for antler mesenchymal cell proliferation and is a critical gene for antler development [85–87]. More broadly, the most divergent windows were often near genes linked to cancer and other biological processes related to development (Table 1), consistent with our understanding of antler growth and function. *LGALS9*, for example is a gene that produces Galactin-9, a well-studied protein expressed on tumour cells [88], and *DMBT1* is a tumour suppressor gene involved in bone cancer [89] that like *IGF1R* has been previously identified as key velvet antler peptide [74]. *MTMR2* (Myotubularin Related Protein 2) has been indicated as a candidate gene for litter size in pelibuey sheep [90] and plays a role in spermatogenesis [91]. Likewise, the qPCR validated *SRP54* gene has been linked to bone marrow failure syndromes and skeletal abnormalities [92]. Thus, we report testable antler-genic links to bone formation, oncogenic pathways, and sexual selection detected from a pooled-GWAS approach.

Using analogous human tissue and cell expression, most of these reported genes appear to have low tissue specificity (*IGF1R*, *MTMR2*, *SRP54*) or are most highly expressed in adaptive immune tissues (*LGALS9*) or in the intestine (*DMBT1*). The specificity for the single cell type expression of these genes all fall into the categories of neuronal cells and glial cells (*IGFR1*, *MTMR2*), glandular epithelial cells (*LGALS9*, *DMBT1*), and germ cells (*MTMR2*, *SRP54*) [93].

The comparison of extreme body size phenotype sequences also revealed an array of genes of interest, but causal inferences are more challenging (see [94]) as traits of this nature, for example human height, involve thousands of SNPs and a multitude of biological pathways [95]. Despite the relatively high heritability of body size in this population [36], there were fewer windows meeting our threshold and no significant GO pathways. Similarly, Taye et al. [96] and Deng et al. [97] detected divergent genes, but no pathways associated with body size.

### Considering a role for transposable elements

Traditionally, masking transposable elements reduces misalignments due to the repetitive nature of their sequences and current limitations with mapping software [98], but also misses a wealth of information that encompasses a large portion of the genome. Our focus was the frequency differences of TE insertions to properly mapped reference sequences, and how these varied between phenotypes. This stems from increasing evidence showing that TE insertions impact gene expression, and thus the variation for a given phenotype [21]. We found 275 highly divergent TE insertions in genic regions from our antler analysis and 104 from the body size analysis. The insertion of these highly variable sequences has the potential to impact gene function and studies are now starting to emerge that both validate insertions [99, 100] and show evidence for positive selection [61, 62]. Our GO pathway analysis that included TE insertions did identify pathways in antler, but not body size, and is an analytical consideration we wanted to highlight to the broader community. Validating the TEs and characterizing their influence on neighbouring genes is also warranted given the high frequency differences between pools.

### Implications for studying quantitative traits in natural populations

Antler and body size are traits that are sexually selected and linked to reproductive success in WTD [35, 101]; they are also traits desired by hunters, managers, and farmers (e.g. antlers for velvet or large body for meat). We have detected highly divergent genomic regions, and thousands genome-wide variants with allele frequency differences between the phenotypic extremes for these traits. Additional lines of evidence, specifically RNA-seq studies (see [84]), should look at the regions identified here, but also consider interactions across the entire genomic, epigenomic, and transcriptomic landscape.

We validated two out of three QTL allele frequencies via qPCR and showed an effect in a mixed model on phenotypes for WTD. While we applied the added stringency of accounting for overdispersion in the chi-square test, our inability to validate one outlier suggests a small effect size (e.g. [36]) and thus requires increased sample size (see [5]), or is a true type I error. Alternatively, epistatic interactions might also be at play [102], but detection requires whole-genome sequencing of many hundreds of individuals thereby defeating the purpose of pooled sequencing. Here, expanded individual genotyping might still reveal the predicted effect, and it is encouraging that approaches for low coverage pooled data are emerging [103].

The unrelated nature of males in our database [36] and null population analyses would support these patterns being inherent to the sampling strategy; however, replicating this approach in other populations would serve as further validation as we anticipate at least a portion of the outlier windows and genes to be shared across the species (e.g. [104]). More broadly, polygenic traits are difficult to characterize under ideal situations, and so the approach presented here is particularly promising for discrete traits, rare alleles and moderate-to-large effect loci (e.g. [17, 18, 25]). As we continue to identify and validate candidate QTLs, it is conceivable that a gene panel could be developed in white-tailed deer and assist in breeding and management programs or assess the effects of artificial selection by trophy hunting; however, until a reasonable amount of phenotypic variation can be attributed to specific QTL, a gene targeted approach for management and breeding of white-tailed deer will remain a challenge.

## Supplementary Information

**Additional file 1.**

**Additional file 2.**

**Additional file 3.**
