# Supplementary material for: Genomic architecture of phenotypic extremes in a wild cervid
Source: BMC Genomics. 2022 Feb 12;23:126. doi: 10.1186/s12864-022-08333-x (PMC8841092; doi:10.1186/s12864-022-08333-x)
Supplement: Supplementary file 1 — Additional file 1. [file 12864_2022_8333_MOESM1_ESM.docx]

**Table S1**. Mean measurements for each sequenced phenotype pool. Only measurements used in the ranking of antler and body phenotypes are presented.

| Phenotype Pool | Age | Body Length | Points | Beam Diameter |
| --- | --- | --- | --- | --- |
| **Large Antler** | 4.96 | 191.49 | 9.06 | 3.27 |
| **Small Antler** | 5.25 | 178.31 | 2.67 | 1.62 |
| **Large Body** | 5.17 | 204.00 | 7.13 | 2.20 |
| **Small Body** | 5.32 | 156.61 | 4.87 | 2.19 |

**Table S2**. Number of reads remaining after each filtering stepped and final genome wide coverage for each pool.

|  | Initial | Deduped | Unique | Coverage |
| --- | --- | --- | --- | --- |
| Large Antler | 733,681,602 | 683,823,491 | 586,588,595 | 33.6 |
| Small Antler | 828,746,267 | 767,420,576 | 652,843,728 | 37.4 |
| Large Body | 794,621,182 | 718,618,777 | 611,366,207 | 34.7 |
| Small Body | 824,962,249 | 728,085,328 | 618,792,299 | 35.3 |

**Table S3**. Primer sequences for the rhAMP genotyping assay used for outlier antler and body SNPs for the RIMS1, SRP54, and LRIF1 genes.

| **Primer** | **Primer Sequence** |
| --- | --- |
| RIMS1_ASP1 | /rhAmp-F/ATCTATTATACTTAGGAAAGTTTCAAAGrGGAAA/GT4/ |
| RIMS1_ASP2 | /rhAmp-Y/ATCTATTATACTTAGGAAAGTTTCAAATrGGAAA/GT4/ |
| RIMS1_LSP | GCTCTTACCCTTTCAATCTCAGAGTGrCTAAA/GT4/ |
| SRP54_ASP1 | /rhAmp-F/ATCCTGAGCCACAGGATATrAAGGT/GT4/ |
| SRP55_ASP2 | /rhAmp-Y/ATCCTGAGCCACAGGATACrAAGGT/GT4/ |
| SRP56_LSP | GCGAGGAAATCAAGTGCTTTTACCAArGAAGA/GT3/ |
| LRIF1_ASP1 | /rhAmp-F/GCCCTTCCCTACTTAAAATGTTTrCATCA/GT2/ |
| LRIF1_ASP2 | /rhAmp-Y/GCCCTTCCCTACTTAAAATGTTCrCATCA/GT2/ |
| LRIF1_LSP | GCTAATCTGCCTTCTCTTTGGTCTrCTCAG/GT4/ |


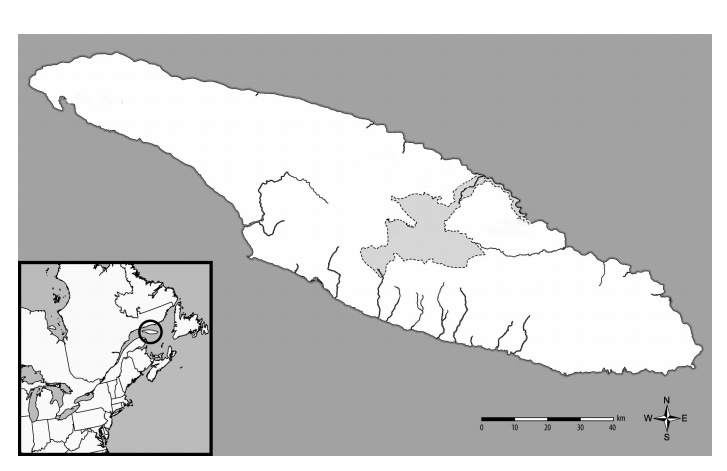


**Figure S1.** A map of Anticosti Island, Quebec where all white-tailed deer samples were acquired between 2002-2014. The grey area reflects Anticosti National Park where no hunting is permitted. The inset represents the larger North American landscape.


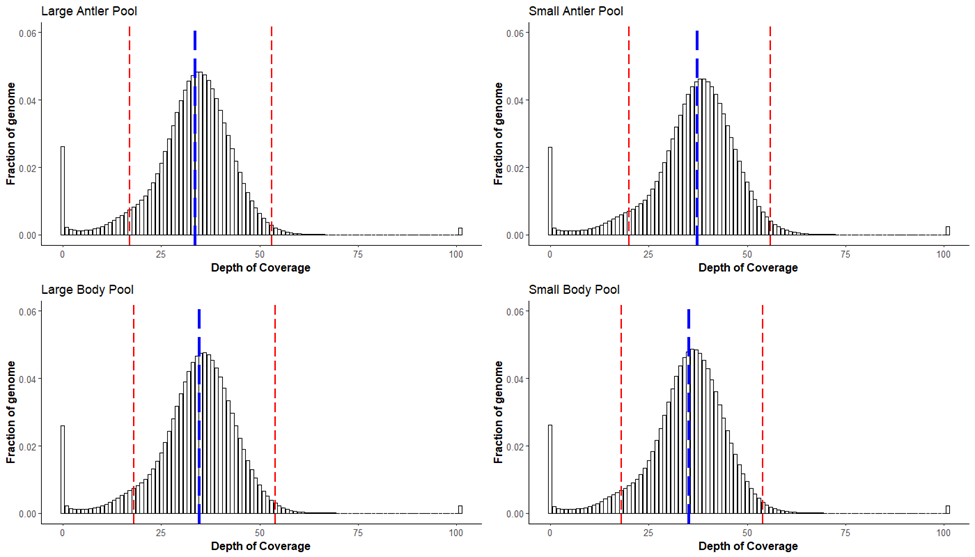


**Figure S2.** Depth of genome coverage throughout the white-tailed deer genome for all pools in our analysis. Dotted blue lines represent the calculated mean depth, while dotted red lines represent the upper and lower cut off values (+/- half the average mode from each pool).


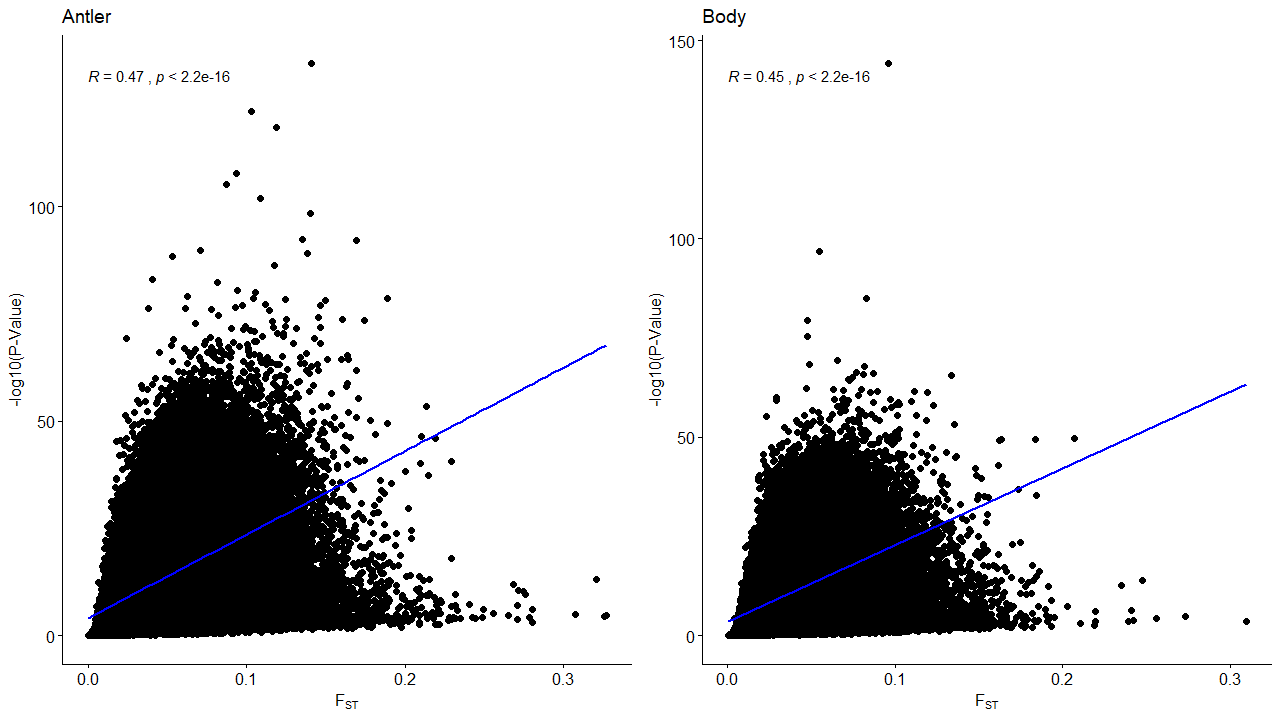


**Figure S3**. Pearson correlation between *F*_ST_ and FET *p*-values in 1000 bp sliding windows (500 bp step size) for all SNPs in both antler (left) and body (right) analysis. Analysis shows a positive correlation (Antler, Pearson’s R = 0.47, p < 2.2e-16; Body, Pearson’s R = 0.45, p < 2.2e-16).


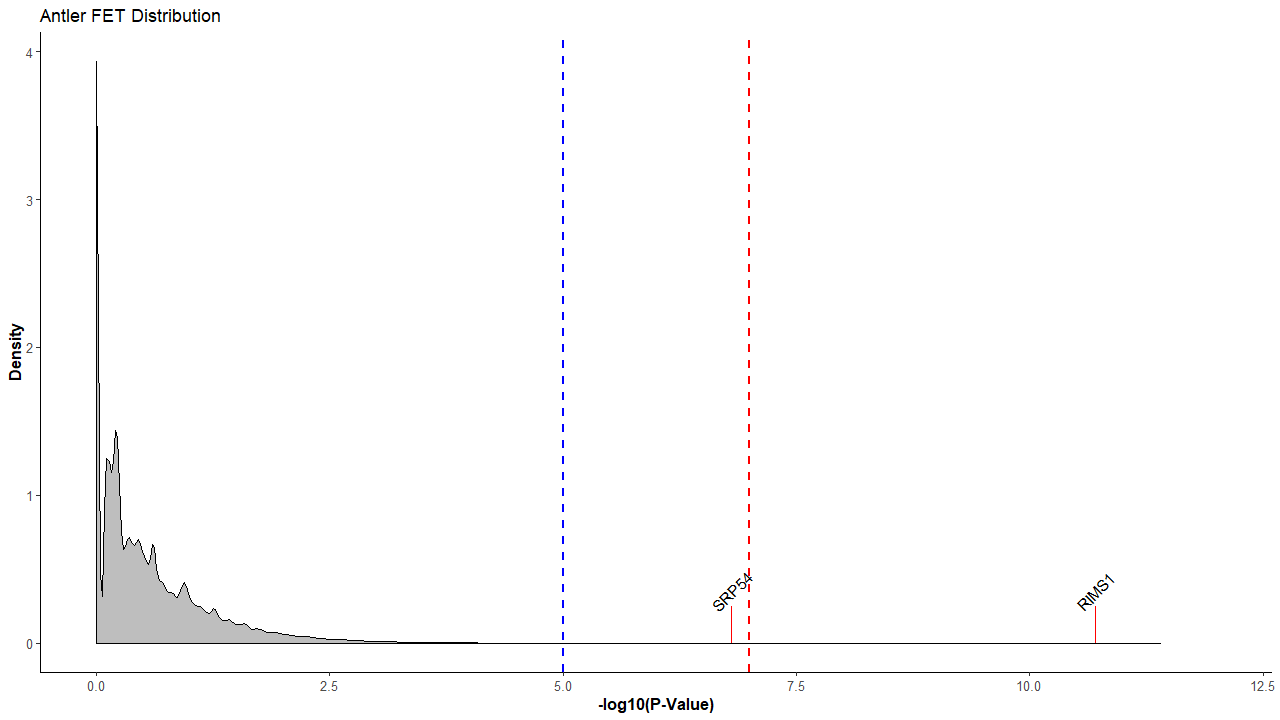


**Figure S4.** Distribution of SNPs based on pairwise fisher’s exact test values (-log10 (p-value) for the antler analysis. Vertical dashed blue line represents the false detection rate and dashed red line represents significance threshold. SNPs used in qPCR validation assays in proximity to the genes *SRP54* and *RIMS1* are highlighted.


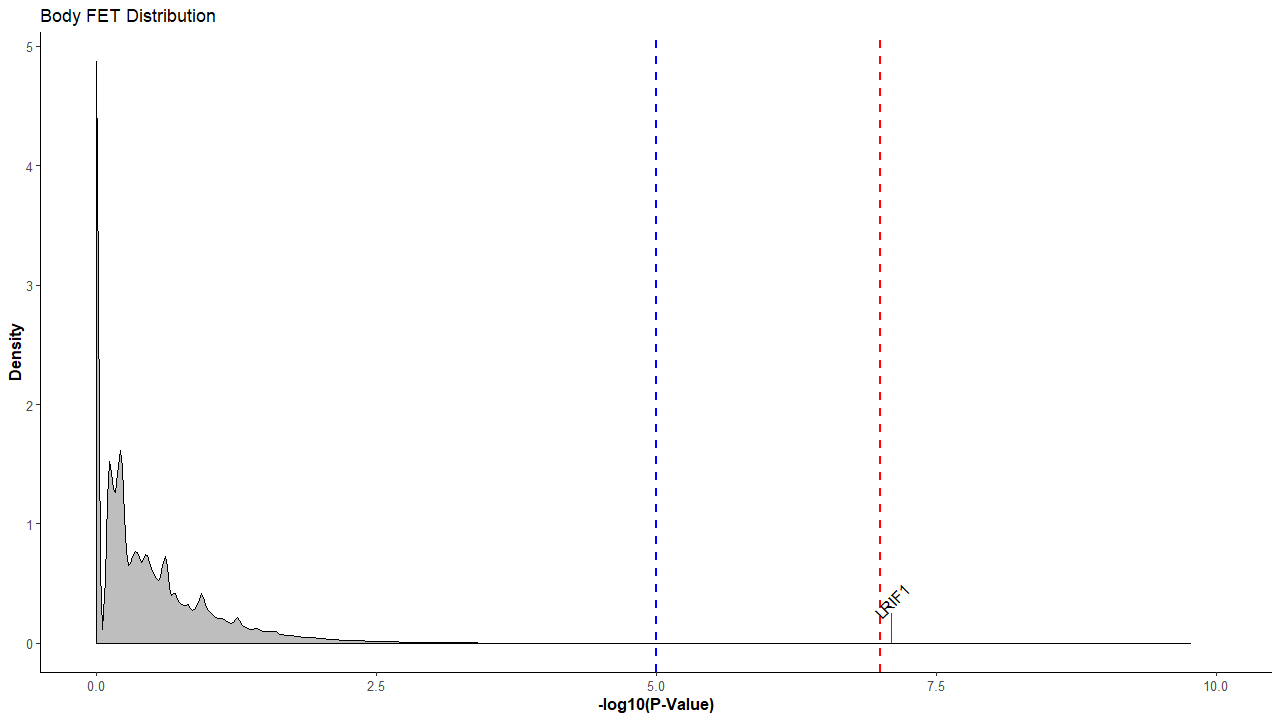


**Figure S5.** Distribution of SNPs based on pairwise fisher’s exact test values (-log10 (p-value) for the body analysis. Vertical dashed blue line represents the false detection rate and dashed red line represents significance threshold. The SNP used in the qPCR validation assays in proximity to the LRIF1 gene is highlighted.


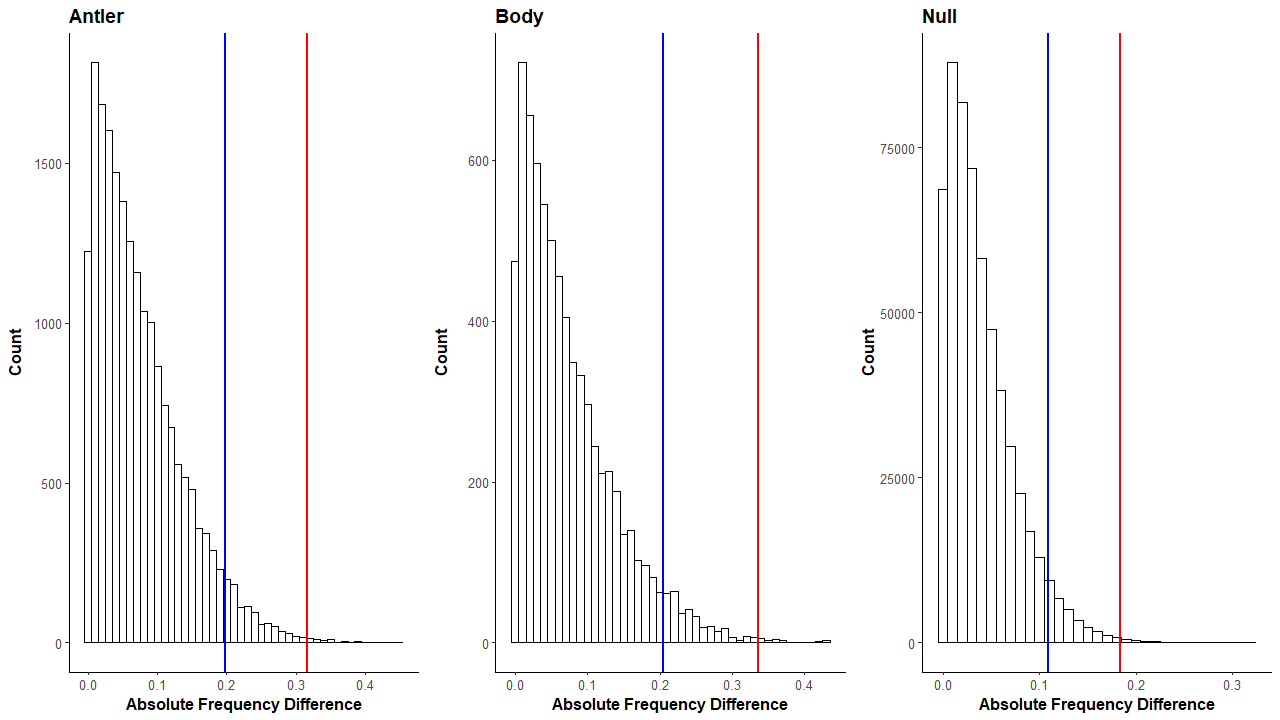


**Figure S6**. Histogram of the absolute frequency difference between transposable elements obtained through comparison of large and small antler pool analysis. Vertical blue bar represents the 95^th^ percentile, and vertical red bar represents the 99^th^ percentile.


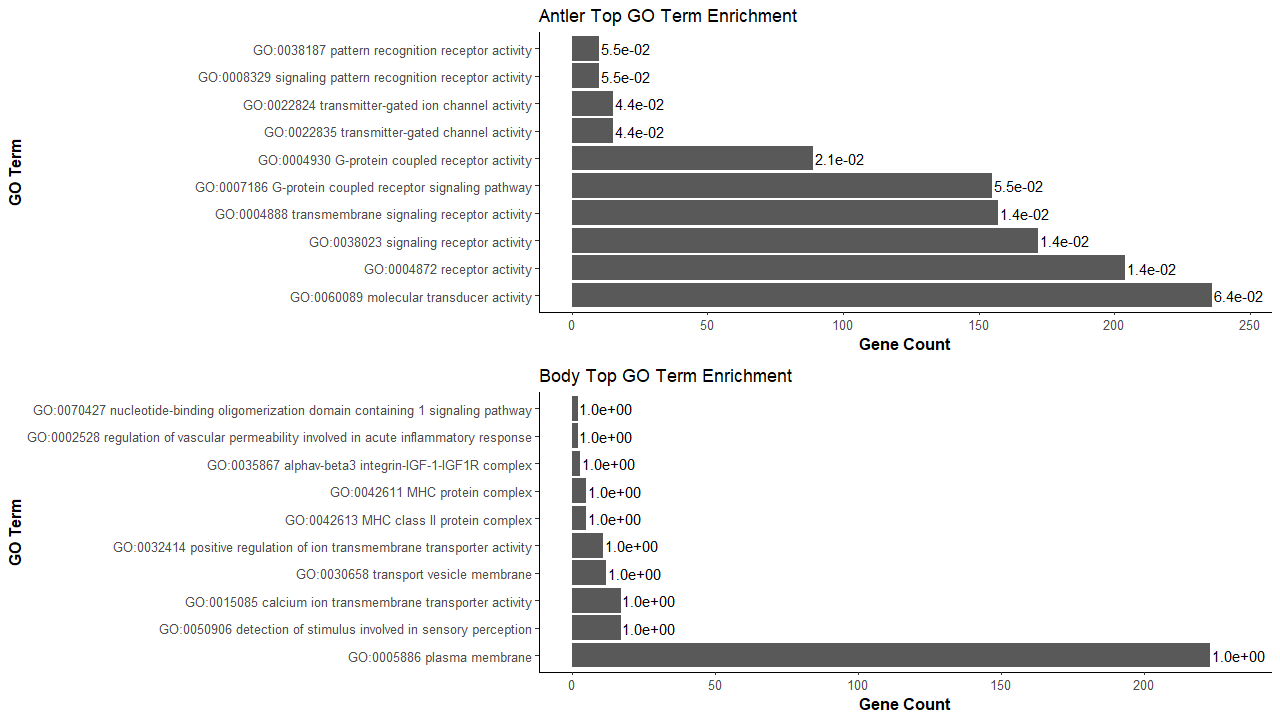


**Figure S7.** Histograms of the top ten enriched GO terms identified through outlier genes in the antler (top) and body (bottom) genome wide association study.

**
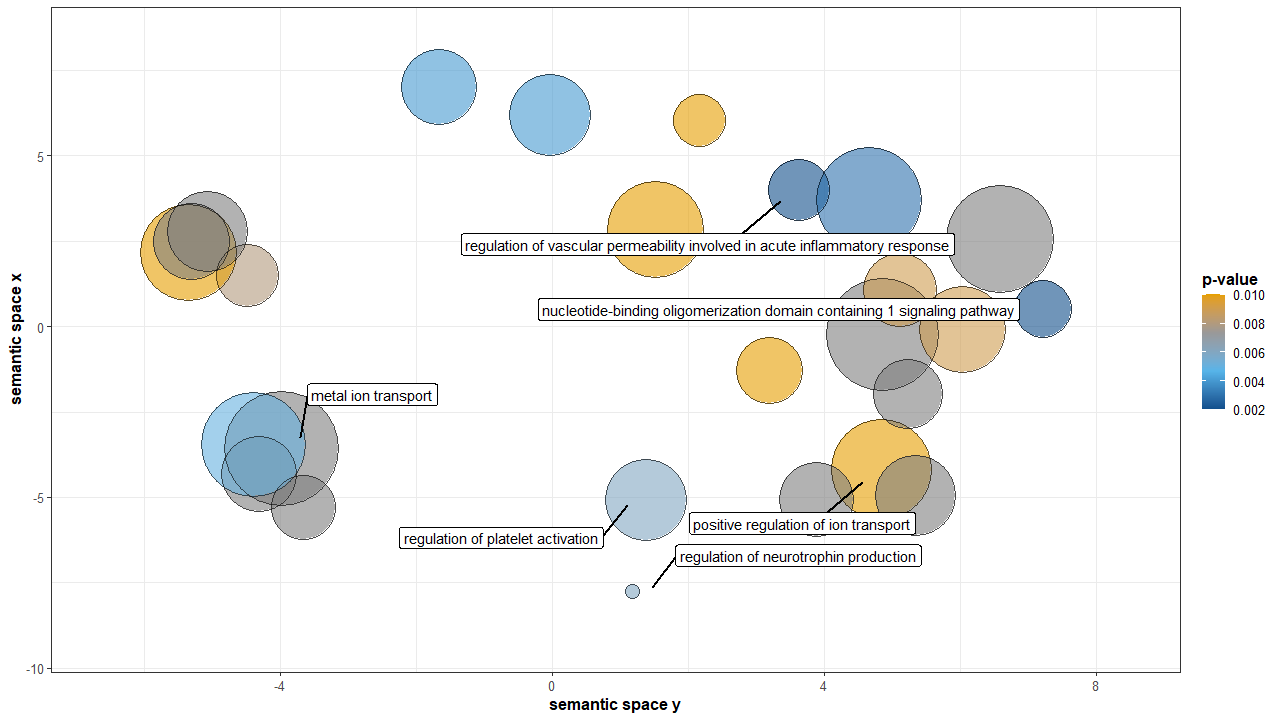
**

**Figure S8.** Body FET analysis of GO terms grouped by semantic similarity. Points are coloured based on significance, with all terms with *p*-values < 0.01 from the output of the Gowinda analysis for gene enrichment being included in the analysis. The size of each point represents the specificity of each term; GO terms for smaller points being more specific, and larger points more general. Only points with a dispensability score < 0.20 are labeled.
